# Antimetabolite Treatment for Pancreatic Cancer

**DOI:** 10.4172/2167-7700.1000137

**Published:** 2014-08-24

**Authors:** Malyn May Asuncion Valenzuela, Jonathan W. Neidigh, Nathan R. Wall

**Affiliations:** 1 Center for Health Disparities Research and Molecular Medicine, Loma Linda University, Loma Linda, California, USA; 2 Department of Basic Sciences, Division of Biochemistry, Loma Linda University, Loma Linda, California, USA

**Keywords:** Chemotherapy, Pancreatic cancer, Gemcitabine, Zebularine, 5FU, Platinum

## Abstract

Pancreatic cancer is a deadly and aggressive disease. Less than 1% of diagnosed patients survive 5 years with an average survival time of only 4–8 months. The only option for metastatic pancreatic cancer is chemotherapy where only the antimetabolites gemcitabine and 5-fluorouracil are used clinically. Unfortunately, efforts to improve chemotherapy regimens by combining, 5-fluorouracil or gemcitabine with other drugs, such as cisplatin or oxaliplatin, have not increased cell killing or improved patient survival. The novel antimetabolite zebularine shows promise, inducing apoptosis and arresting cellular growth in various pancreatic cancer cell lines. However, resistance to these antimetabolites remains a problem highlighting the need to discover and develop new antimetabolites that will improve a patient’s overall survival.

## Introduction

In the United States, pancreatic cancer is the 4^th^ leading cause of cancer death aggressively and silently attacking the patient [1–3]. Pancreatic cancer is only identified in more advanced stages when the patient is symptomatic, as there are no screening tests for this disease [4]. At the time of diagnosis, approximately 85% of the patients have advanced pancreatic cancer resulting is a short median survival time of 4–8 months where less than 1% survive more than 5 years [5,6]. Currently, the best treatment is surgical resection where approximately 20% of patients increase their life span by approximately 2 years [7]. For metastatic pancreatic adenocarcinoma, chemotherapy using gemcitabine (GEMZAR) is currently the only first-line FDA approved treatment [8]. Antimetabolite drugs are designed to stop DNA replication and normal cellular metabolic processes by different mechanisms and have been investigated for almost 70 years [9,10]. Currently, efforts to improve the treatment for metastatic pancreatic cancer explore using combinations of therapeutic agents as well as searching for new antimetabolite drugs. This review will discuss the different antimetabolite agents used to treat pancreatic cancer, both clinically approved and experimental, their mechanisms of action, and therapy resistance.

### 5-Fluorouracil

**Figure 1:**
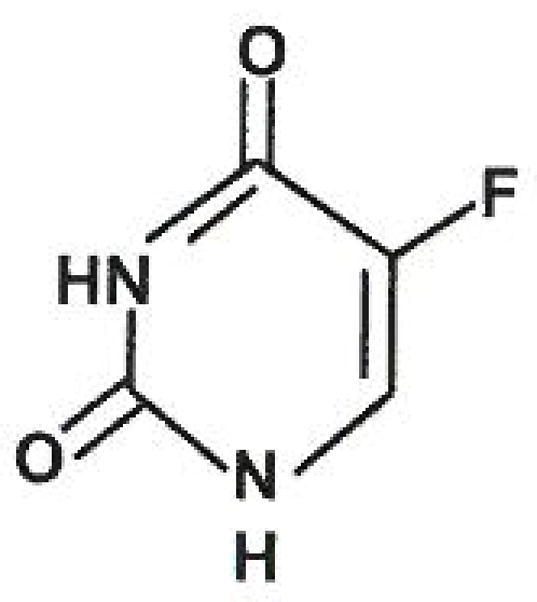
Structure of 5FU with the fluor group in carbon 5-position. 5FU is a pyrimidine analog drug whose mechanism of action is through irreversible inhibition of thymidylate synthase (TS). Clinically is have been used in the treatment of anal, breast, colorectal, esophageal, stomach, pancreatic and skin cancers.

The pyrimidine 5-fluorouracil (5FU) has been under investigation for the treatment of human cancers since 1954 when it was observed that uracil is utilized more efficiently by tumor cells than normal cells [11]. The knowledge that fluorine substitutions of hydrogen in metabolites often resulted in a toxic compound inspired the design of 5FU (Figure 1) and testing as a tumor-inhibiting compound [11–13]. Since its discovery, 5FU has been used as a treatment for many solid tumors such as colon, breast, head and neck cancers, and advanced pancreatic cancer. For 20 years, 5FU was regarded as the only effective drug against advanced pancreatic cancer. However, despite numerous efforts to improve therapy outcomes, the best response rate was approximately 20% [12,14,15].

### Mechanism of action

**Figure 2:**
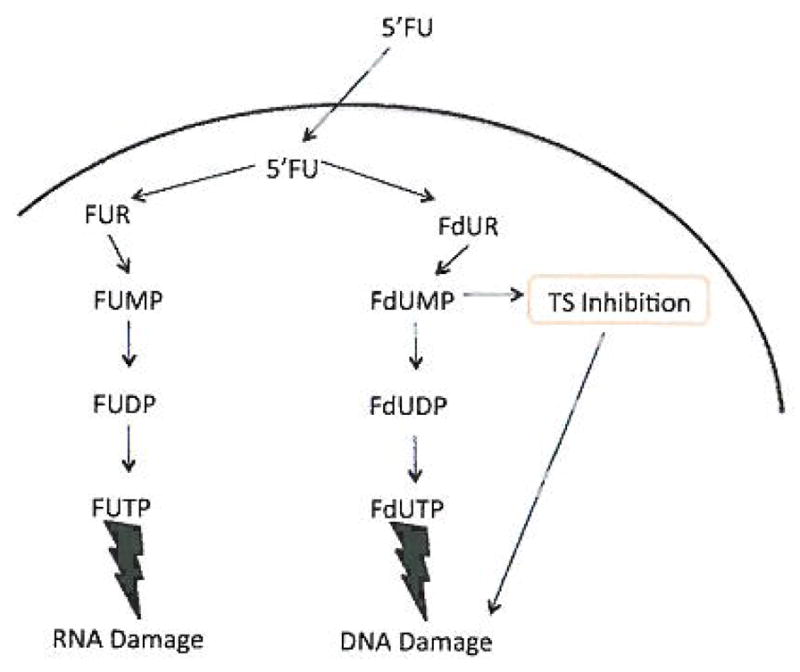
Mechanism of 5FU leading to RNA and DNA damage. Thymidylate synthase inhibition is the main mechanism of action of 5FU through its active metabolite FdUMP. Synthesis of the pyrimidine thymidine, which is required for DNA synthesis, is the result of blocking thymidylate synthase. Thymidylate synthase methylates deoxyuridine monophosphate (dUMP) to for thymidine monophosphate (dTMP). The use of 5FU in cancer causes there to be a reduction leading to a scarcity of dTMP so that rapidly dividing cancer cells die from a lack of thymine.

Like uracil, 5FU is salvaged to form 5-fluorouridine and then phosphorylated by nucleoside and nucleotide kinases as well as reduced by ribonucleotide reductase forming three different active metabolites (Figure 2). After incorporation of 5-fluorouridine triphosphate (FUTP) into cellular RNA, RNA processing and post-transcriptional modification can be inhibited [15,16]. During DNA synthesis, 5-fluoro-2′-deoxyuridine monophosphate (FdUMP) inhibits thymidylate synthase resulting in an imbalanced pool of deoxynucleotide triphosphates, particularly decreased deoxythymidine triphosphate (dTTP) and increased deoxyuridine triphosphate (dUTP). Absent dTTP, stalled DNA polymerases can incorporate 5-fluorodeoxyuridine triphosphate (FdUTP) or dUTP which are subsequently recognized as damaged DNA setting up a futile cycle of misincorporation and repair [15,16]. When DNA damage exceeds a cells ability to repair misincorporated FdUTP or dUTP, single strand and double strand breaks accumulate favoring cell death. Given these cellular actions of 5FU, its toxicity is generally considered a function of transport into the cell and metabolism to active metabolites, particularly FdUMP, while resistance occurs when 5FU metabolism is decreased or DNA repair is efficient.

#### Resistance

One mechanism of 5FU resistance may result from high levels of thymidylate synthase expression in pancreatic cancer patients. Head and neck [17] and gastric [18] cancer patients with low tumoral thymidylate synthase expression exhibited increased sensitivity to 5FU treatment, while a lack of response was seen in advanced colorectal patients [19] with high thymidylate synthase expression. Interestingly, the opposite was observed where node-positive breast [20] and Dukes’ B and C rectal [21] cancer patients with high expression levels of thymidylate synthase responded well to 5FU therapy. It is not currently known why this phenomenon was seen, but 5FU therapy-outcome may be associated with the tumor type that is being treated or with the biome of stress-associated molecules expressed and/or induced. One retrospective study of pancreatic cancer patients found that 5FU resulted in longer survival for patients with low thymidylate synthase expression [22]. Further translational studies are needed to better understand the role of thymidylate synthase expression and therapy outcome [10,16]. These and other studies on the mechanism of resistance continue and may prove instrumental in understanding resistance leading to better therapeutic design and combinations.

An additional mechanism of resistance is decreased expression 5FU transport into pancreatic cancer cells. In human pancreatic cancer cell lines, the sensitivity to 5FU directly correlated with the expression level of the human equilibrative nucleoside transporter 1 (hENT1) [23]. However, increased median survival time in pancreatic cancer patients treated with 5FU was not significantly different [24]. Additional studies are needed to understand the differences in resistance to 5FU in cell lines as opposed to pancreatic cancer patients.

### Gemcitabine

Gemcitabine (2′, 2′-difluoro-2′deoxycytidine, dFdC) was originally considered as an antiviral drug [25], but was later shown to demonstrate anti-cancer activity in both *in vivo* and *in vitro* models of solid and hematological cancers [14,25,26]. Today, gemcitabine is the only FDA approved single chemotherapy agent against metastatic pancreatic cancer, showing a better 1-year survival rate, median survival, and clinical benefit when compared to 5FU [8].

#### Mechanism of action

**Figure 3:**
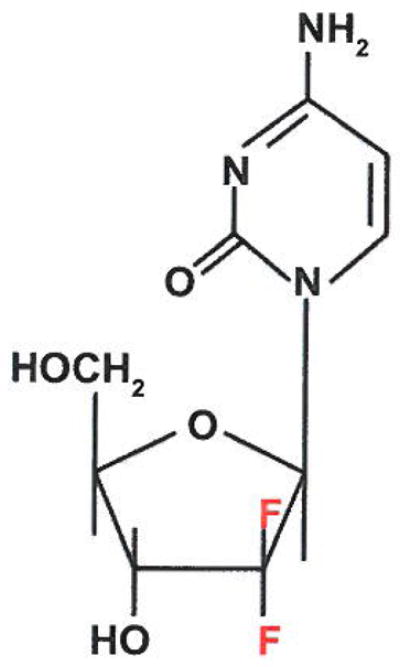
Gemcitabine is a nucleoside analog in which the hydrogen atoms on the 2′ Carbon of deoxycytidine are replaced by fluorine atoms. Like other analogues of pyrimidines, the triphosphate analogue of gemcitabine replaces the important cytidine building block of nucleic acids during DNA replication arresting tumor growth and resulting in apoptosis. Gemcitabine has been used to treat various carcinomas including lung, pancreatic, bladder and breast cancers. It is being investigated for the possible use against esophageal cancers and lymphomas.

**Figure 4:**
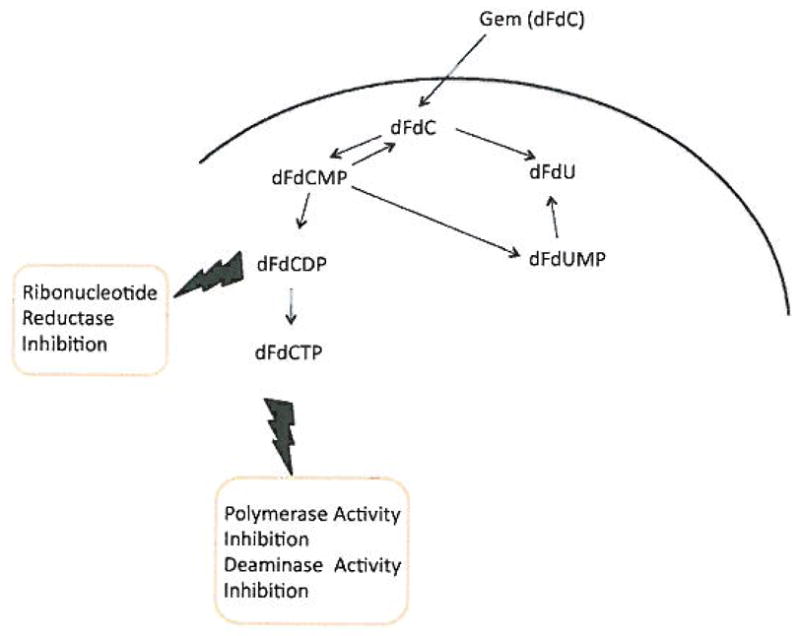
The broad spectrum mechanism of action of Gem, depending on its phosphorylation state, can inhibit Ribonucleotide Reductase, Polymerase and Deaminase activities. Once these enzymes are irreversibly inhibited, the cell cannot produce the deoxyribonucleotides required for DNA replication and repair and the cell dies via apoptosis.

Gemcitabine is a 2′-deoxycytidine analogue with fluorine substituted for hydrogen at the 2′ position of the furanose ring (Figure 3). Gemcitabine is a broad-spectrum agent, which has different mechanisms of action, depending upon its phosphorylation state (Figure 4) [8,25]. Uptake of Gem into the cell uses both human equilibrative nucleoside transporters (hENTs) and human concentrative nucleoside transporters (hCNTs) [27,28]. Inside the cell, gemcitabine is phosphorylated by deoxycytidine kinase into gemcitabine monophosphate (dFdCMP), which is further converted into its active di- and triphosphate (dFdCDP and dFdCTP) states by nucleotide kinases [29]. Ribonucleotide reductase is inhibited by dFdCDP leading to a reduction in dCTP levels. Reduced dCTP lessens the negative feedback regulation of deoxycytidine kinase and favors the efficient phosphorylation of gemcitabine [30]. The cytotoxic activity of gemcitabine leading to apoptosis is mainly the result of its triphosphate form. DNA polymerase activity is inhibited when dFdCTP is incorporated into the DNA strand leading to a termination of the DNA chain synthesis and single strand breakage [31–33]. Consequently, a depletion of dCTP levels, due to inhibition of ribonucleotide reductase activity, results in the competition of dFdCTP with dCTP leading to an increased incorporation of dFdCTP into the DNA strand [30]. In addition, high intracellular levels of dFdCTP also strongly inhibited dCMP deaminase activity, by directly inhibiting the deaminase as well as indirectly because of the decreased Dctp:dTTP ratio [34].

#### Resistance

It has been shown *in vitro* that low levels of hENT1, leading to limited gemcitabine intracellular uptake, is a mechanism of chemoresistance [23,35,36]. In pancreatic cancer patients, the levels of hENT1 were recently observed to correlation with overall median survival time, where patients with higher levels of hENT1 have better survival rates [24]. Further mechanisms of resistance to gemcitabine observed in cell lines from multiple cancer types resulted from decreases in deoxycytidine kinase activity and increased ribonucleotide reductase activity [37]. Implications for pancreatic cancer patients regarding activity and expression of these enzymes, however, are still unknown [38].

### Platinum

**Figure 5:**
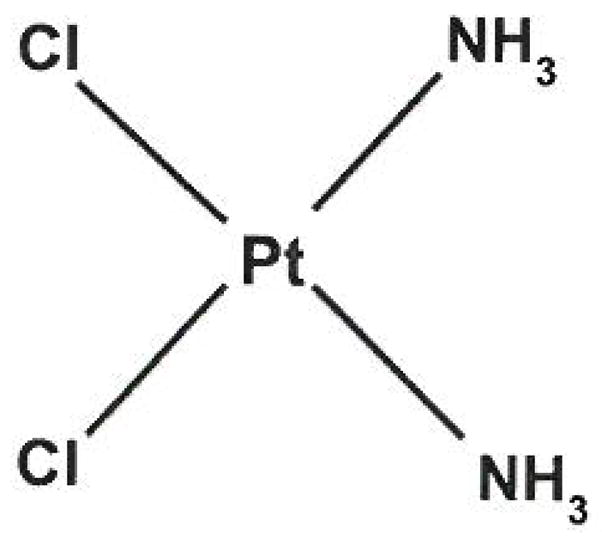
Cisplatin has two chloride ions and two amine groups attached to the center platinum ion. Cisplatin has been used to treat various cancers which include sarcomas carcinomas of the lung and ovary, lymphomas and germ cell tumors and is especially effective in treatment of testicular cancer.

**Figure 6:**
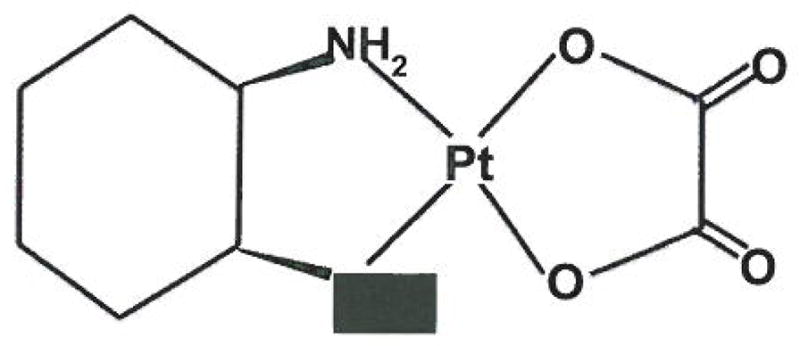
Similar to Cisplatin, Oxaliplatin contains a doubly charged platinum ion in the center. It, however, contains diamnocyclohexane and carboxylate compounds. These platinum complexes bind to and crosslink DNA *in vivo* which triggers apoptosis.

Platinum agents are used today in combination therapy regimes with gemcitabine as second line chemotherapy for metastatic pancreatic cancer. Cisplatin (dis-diamminedichloroplatinum, CDDP, PtCl2(NH3)2) is shown in Figure 5 and is an inorganic platinum complex composed of a doubly charged platinum ion, and four ligands-two chloride ions and two amines. Cisplatin is a potent chemotherapy drug discovered in the 1960’s. It is widely used today against a variety of tumors including head and neck, non-small cell lung, stomach and bladder cancers, non-Hodgkin’s lymphoma and sarcomas [39,40], Oxaliplatin (trans-l-1,2-diaminocyclohexane oxalatoplatinum) (Figure 6) is a new platinum agent that is more potent *in vitro* and has a better toxicity profile compared to cisplatin, as it only needs a small number of DNA adducts to attain the same cytotoxicity profile as cisplatin. In preclinical studies, oxaliplatin shows efficacy in a number of cancer cell lines, which also includes cell lines that are cisplatin resistant [41,42]. This provides hope that with minor modification of these platinum compounds, not only will efficacy increase, but resistance will decrease as well.

#### Mechanism of action

Once taken up into the cells, the chloride ions are lost and replaced with water molecules transforming cisplatin into a reactive species. Loosely bound, the water molecules easily fall off, exposing the platinum ion which readily forms bonds with DNA bases, forming DNA-DNA cross-links and DNA-protein cross-links. These cross-links between bases are usually formed at sites where adenosine and guanine are adjacent on the same DNA strand. It has been speculated that the cis-geometry of cisplatin is important to its anti-tumor activity, as the trans-isomer of cisplatin, transplatin, is inactive [43]. Unlike 5FU, cisplatin chemotherapy arrests cells at the G1, S or G2-M phase of the cell cycle, making this drug efficient in killing cells that are in all stages of the cell cycle [39,44–46].

In oxaliplatin, the two amines and two chloride ions of cisplatin are replaced with diaminocyclohexane and carboxylate compounds, respectively (Figure 6). Similar to cisplatin, once inside the cell, the carboxylate compound is displaced, transforming oxaliplatin into a reactive compound that forms DNA intra-strand cross-links, DNA interstrand cross-links, and DNA-protein cross-links [45]. DNA lesions induced by intrastrand cross-links are formed when the drug binds to two adjacent guanine bases, and to a lesser extent, to adjacent adenosine and guanine bases. Binding of the mismatch repair protein complex to the DNA becomes more difficult due to the conformation of adducts, which may result in poor repair of the lesion. Oxaliplatin has been reported to inhibit TS activity, much like 5FU [44,45].

#### Resistance

There are several mechanisms whereby tumor cells become resistant to both cisplatin and Oxaliplatin. The toxicity of cisplatin and oxaliplatin is reduced in cells with an efficient repair of damaged DNA where enzymes involved in nucleotide excision repair remove the platinum-DNA adducts [39]. The relationship between enhanced platinum resistance, a decrease in drug sensitivity, and increased DNA repair protein levels has been described [39,47,48]. Another mechanism is through a decrease in intracellular platinum concentration resulting from a reduction in drug uptake and an increase of platinum expulsion out of the cell or detoxification by glutathione and metallothionein and an increased level of glutathione and metallothionein has been shown in some cases to correlate with cisplatin resistance [49]. This resistance is not due to only one mechanism, but on a variety of mechanisms targeting various systems [39,44,45], The mechanisms of resistance for cisplatin and oxaliplatin differ from the mechanisms of resistance for gemcitabine resulting in a benefit from combining these agents in a therapeutic regimen.

### Combination therapy with platinum agents

Cisplatin and oxaliplatin are not used as single agents against pancreatic cancer, but rather, in combination with either gemcitabine or 5FU when treatment with gemcitabine alone has failed. There have been multiple studies on the effects of cisplatin used in combination with gemcitabine. One phase III study showed that compared to patients treated with gemcitabine alone, the overall median survival and progression-free survival of patients on the Gemcitabine-cisplatin combination therapy improved, but did not reach statistical significance [50]. Furthermore in another study, comparable results in patients treated with Gemcitabine alone or in combination with cisplatin were observed [46]. However, they also noted that the combination therapy was more toxic than gemcitabine alone. Nevertheless, studies do show favor for a Gem-cisplatin combination, where disease progression and the median 1-year event-free survival is encouraging [42]. Oxaliplatin has been used in combination with both Gemcitabine and 5FU. One study has shown that patients with inoperable pancreatic cancer tolerated the combination of Gemcitabine with oxaliplatin well and was recorded to be highly effective [51] while a phase II trial showed moderate activity [41]. When in combination with 5FU, clinical benefits were recorded and toxicity levels were acceptable [52]. These platinum agents, when combined with Gemcitabine or 5FU, may be a promising treatment regime for pancreatic cancer patients.

### Zebularine

**Figure 7:**
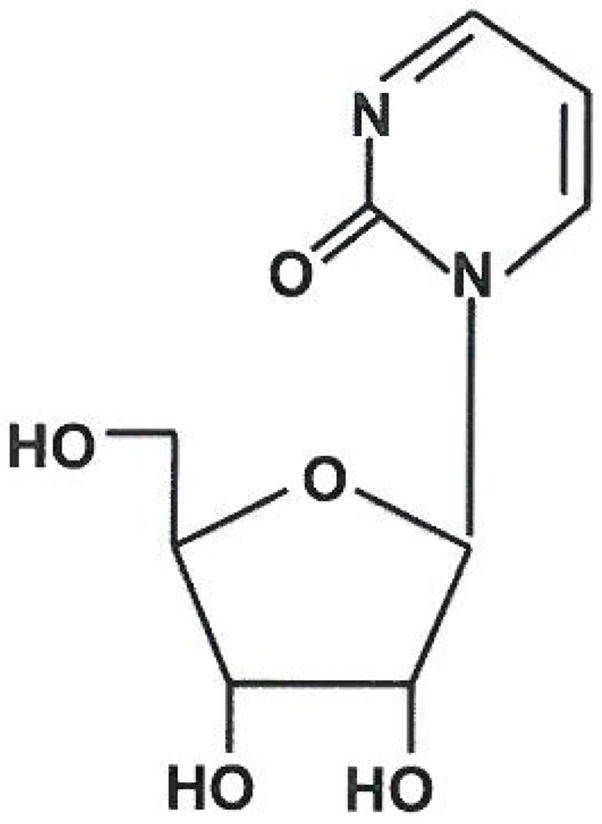
Zebularine’s structure includes a 2(1H)-pyrimidinone ring. It is a nucleoside analog of cytidine and works by inhibiting cytidine deaminase by binding to the active site as a covalent hydrates. It has also been shown to inhibit DNA methylation and tumor growth in vivo and in vitro. Though entirely experimental at this time, it has been suggested that it could be used as a chemoprevention agent or even in epigenetic therapy.

Epigenetic changes accompany pancreatic tumorigenesis as well as the acquisition of resistance to chemotherapy [53,54]. Therapeutic agents that alter the epigenetic state of pancreatic cancer cells are under investigation as cytotoxic agents as well as agents to reverse acquired resistance to first-line agents. Lacking an amino group on the C-4 position of the pyrimidine ring, zebularine ((1-β-D-Ribofuranosyl)-2(1H)-pyrimidinone), a cytidine analogue (Figure 7), was originally developed as a cytidine deaminase inhibitor. It is also a novel DNA methytransferase (DNMT) inhibitor and unlike other DNMT inhibitors, zebularine is more stable in aqueous solution and is less toxic *in vitro* and *in vivo* [55–57]. Continuous exposure of numerous cancer cell lines to zebularine slowed tumor cell growth as compared to normal human fibroblast cell lines indicating its promise as a chemotherapy agent for cancer treatment [58].

#### Mechanism of action

Once inside cells, zebularine is phosphorylated by uridine-cytidine kinase. Nucleotide kinases phosphorylate zebularine monophosphate to form zebularine triphosphate, which is then incorporated into DNA. The 2(1H)-pyrimidinone ring is important as its incorporation into the DNA strand leads to DNMT1 depletion and DNA methylation inhibition. When zebularine replaces cytosine in a CpG dinucleotide and a DNA methyltransferase attempts to methylate zebularine, an irreversible covalent complex is formed thus inhibiting DNA methylation [58]. In a transgenic mouse model of breast cancer, zebularine slows tumor growth and induces cell death by both necrosis and apoptosis [55]. Other studies show that zebularine decreases levels of DNMT1, DNMT3a, and DNMT3b in breast cancer cell lines [59] as well as DNMT1 and partially DNMT3b in bladder cancer cells [58]. A reduction in DNMT1 and DNMT3b was also shown in the mammary tumors in transgenic mice [55]. The growth inhibition property of zebularine may be due to drug incorporation into the DNA. However, the amount of zebularine in DNA was low in normal cells and growth was minimally affected, while the opposite was seen in cancer cells [58]. Understanding incorporation aspects of this agent may prove useful in developing more effective analogues.

### Zebularine and pancreatic cancer

Studies have shown that zebularine effectively slows cellular growth in CFPAC-1, a pancreatic cancer cell line, by inducing the p21 and/or p16 genes [58]. The p21 protein in response to DNA damage, directly stops DNA replication and arrests cellular growth. They have also shown a decrease in DNMT1 through the incorporation of the 2(1H)-pyrimidinone ring, as stated above [58]. In addition, studies also showed that zebularine, as a single agent, induced apoptosis and growth arrest by inhibition of DNMT1 in three pancreatic cancer cell lines: YAP C, DAN G and Panc-89 [60]. Though there are minimal studies showing the potential use of zebularine in pancreatic cancer, initial reports show promise for the use of zebularine in treating pancreatic cancer. More studies, however, are needed to fully test the full potential of zebularine *in vivo*.

## Conclusion

The only effective treatment option available for patients with advanced metastatic pancreatic adenocarcinoma remains the antimetabolite gemcitabine. Despite efforts to improve therapy regimens by using 5FU or Gem in combination with alkylating agents, the prognosis for treating metastatic pancreatic cancer remains bleak. Therefore, it is imperative to continue studying and developing novel antimetabolite agents, such as zebularine, to improve treatment options and improve overall survival rates.
